# Malaria Prevalence in Endemic Districts of Bangladesh

**DOI:** 10.1371/journal.pone.0006737

**Published:** 2009-08-25

**Authors:** Ubydul Haque, Syed Masud Ahmed, Shahed Hossain, Mamun Huda, Awlad Hossain, Mohammad Shafiul Alam, Dinesh Mondal, Wasif Ali Khan, Mohammod Khalequzzaman, Rashidul Haque

**Affiliations:** 1 International Center for Diarrhoeal Disease Research Bangladesh, Mohakhali, Dhaka, Bangladesh; 2 BRAC, BRAC Centre, Dhaka, Bangladesh; University of Oxford, United Kingdom

## Abstract

**Background:**

Following the 1971 ban of DDT in Bangladesh, malaria cases have increased steadily. Malaria persists as a major health problem in the thirteen south-eastern and north-eastern districts of Bangladesh. At present the national malaria control program, largely supported by the Global Fund for AIDS, Tuberculosis and Malaria (GFATM), provides interventions including advocacy at community level, Insecticide Treated Net (ITN) distribution, introduction of Rapid Diagnostic Tests (RDT) and combination therapy with Coartem. It is imperative, therefore, that baseline data on malaria prevalence and other malaria indicators are collected to assess the effectiveness of the interventions and rationalize the prevention and control efforts. The objective of this study was to obtain this baseline on the prevalence of malaria and bed net use in the thirteen malaria endemic districts of Bangladesh.

**Methods and Principal Findings:**

In 2007, BRAC and ICDDR,B carried out a malaria prevalence survey in thirteen malaria endemic districts of Bangladesh. A multi-stage cluster sampling technique was used and 9750 blood samples were collected. Rapid Diagnostic Tests (RDT) were used for the diagnosis of malaria. The weighted average malaria prevalence in the thirteen endemic districts was 3.97%. In five south-eastern districts weighted average malaria prevalence rate was 6.00% and in the eight north-eastern districts weighted average malaria prevalence rate was (0.40%). The highest malaria prevalence was observed in Khagrachari district. The majority of the cases (90.18%) were *P. falciparum* infections. Malaria morbidity rates in five south-eastern districts was 2.94%. In eight north-eastern districts, morbidity was 0.07%.

**Conclusion and Significance:**

Bangladesh has hypoendemic malaria with *P. falciparum* the dominant parasite species. The malaria situation in the five north-eastern districts of Bangladesh in particular warrants urgent attention. Detailed maps of the baseline malaria prevalence and summaries of the data collected are provided along with the survey results in full, in a supplemental information

## Introduction

Malaria is estimated to be directly responsible for around one million deaths annually worldwide [1]. The morbidity and mortality burden caused by malaria are responsible for nearly 3% of the world's DALYs [2]. Even though Africa accounts for 90% of the mortality burden for malaria, South-east Asia still suffers considerable mortality and morbidity. Malaria is a major public health problem in Bangladesh. Of the 11 countries of the World Health Organization South East Asian Regional Office, ten countries including Bangladesh are malaria endemic.

Due to the frequent use of DDT by the Malaria Eradication program of the then Government of East Pakistan, malaria was mostly under control before 1971 [3]. After the independence of Bangladesh from Pakistan, DDT was banned in 1985 and the number of malaria cases began to increase. Since the incidence of malaria in the eastern regions was low and there was a lack of adequate funds and programs, no control efforts maintained in the malaria endemic areas of Bangladesh. Without these control efforts, malaria cases started to increase and became epidemic in the 1990s [3], [4]. In the late 1990s, more than 500 deaths were reported with 70,000 laboratory-confirmed cases and 900,000 clinical cases of malaria in Bangladesh [5].

The number of malaria cases in Bangladesh fluctuates seasonally. The majority of these cases occur in the thirteen districts close to and/or bordering India and Myanmar. These thirteen districts, out of the 64 administrative districts of Bangladesh, are recognized as malaria endemic. Ninety eight percent of the malaria case reports come from these thirteen districts. Three out of these thirteen districts, Bandarban, Khagrachari and Rangamati, collectively known as the Chittagong Hill Tracts (CHT) districts, report the highest incidence of malaria within the country. These thirteen districts are difficult to reach due to the hilly terrain and therefore have inadequate passive surveillance and information systems resulting in poor reporting of malaria cases by the Ministry of Health, Government of Bangladesh [6], [7].

In 2006, the Global Fund for AIDS, TB and Malaria (GFATM), awarded Bangladesh USD 39.6 million to support the national malaria control program. These funds were used by the national malaria control program to start advocacy at community level, ITN distribution, introduction of rapid diagnosis test (RDT) and to introduce combined therapy with coartem. In the absence of baseline information, these resources could not be equitably distributed among the population. Furthermore, these data would be needed for future comparisons to assess the effectiveness of these programs. Therefore in 2007, BRAC and ICDDR,B carried out this first ever malaria prevalence survey of Bangladesh, the results of which are presented here.

## Methods

### Objectives

Our primary objective was to assess the prevalence of malaria in thirteen malaria endemic districts of Bangladesh. In addition to the prevalence survey, we investigated the use of bednets in endemic regions.

### Study Area

The research was carried out in seventy Thanas (sub districts) of thirteen malaria endemic districts of Bangladesh. According to the Ministry of Health, Government of Bangladesh, 98% of the cases are reported from these Thanas of Bangladesh [8]. These thirteen districts are divided into two parts. Eight north-eastern districts share a common border with India. Five south-eastern districts situated in the south-eastern part of Bangladesh. Three of these districts share the common border with India and Myanmar and are known as Chittagong Hill Tracts (CHT) Districts. These three districts area is mostly hilly, forested and thus relatively inaccessible.

### Sample size calculation

We anticipated the malaria prevalence in our eight north-eastern endemic districts to be around 2%, while in the five south-eastern endemic districts it would be more than 3%. Given the design of the study as a cluster survey, the sample size had to incorporate these expectations [10]. The sample size was calculated using web-based software C-Survey 2.0 based on the conservative estimates of malaria prevalence and the design effect. Sample size was estimated assuming the lowest estimate of malaria prevalence at 2% with a precision of 1.5%, at 95% confidence interval with a design effect of two. Thus, 750 individuals from 750 households were taken in each district for this study. Totally 9,750 individual samples from thirteen malaria endemic districts were collected for this study.

### Sampling

**Figure 1:**
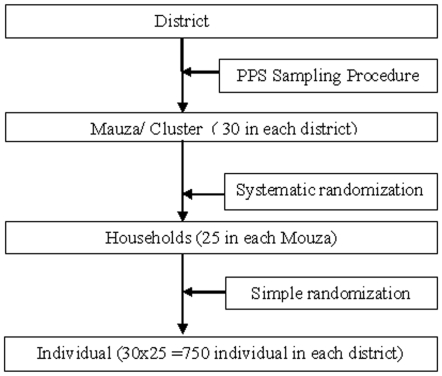
Shows study procedure and sampling.

A three stage cluster sampling technique was employed using population figures from the 2001 census. City Corporations and towns were excluded from this survey because they are urban and of extremely low malaria risk. For each of the thirteen districts, all Mauzas (Mauzas are the lowest administrative unit of Bangladesh, bigger than village and have a polygon boundary) were listed alphabetically and 30 Mauzas were selected using a probability proportional to size (PPS) sampling procedure [11]. These Mauzas were the primary sampling unit of the survey. Twenty-five households were selected using systematic randomization from each Mauzas/cluster (Figure 1). All population in a cluster was eligible to participate in the survey. Information about bed net use was obtained from the head or spouse (or a knowledgeable member in their absence) of the selected household. For prevalence (parasitological) survey, one household member from each household was randomly chosen.

### Ethical considerations

The protocol was reviewed and approved by the research review committee and the ethical review committee of ICDDR,B. The probability and magnitude of harm or discomfort anticipated in the proposed research are not greater in and of themselves than those ordinarily encountered in daily life or during the performance of routine physical, psychological examinations or tests. The study is therefore expected to be minimal risk. Written consent was obtained from the participants.

### Recruitment, training and deployment of the interviewers

The survey team comprised of experienced interviewers and their supervisors. A five-day intensive training was organized for the interviewers consisted of didactic lectures, mock interviews, role play and field practice at community level. Each team typically consisted of four members including one person trained in malaria microscopy. The training was organized by the experts from BRAC and ICDDR,B prior to the start of the survey.

### Field operation

The malaria prevalence survey was conducted during September, October and November 2007. In each of the selected mauza (“clusters”), the study team drew a map. The field team selected households systematically, as they moved from the center or periphery, following a designated path determined by the “spin the bottle” methodology [11].

All members of the household were listed. Only one individual from a household was enrolled into this study using a simple randomisation procedure. Informed consent was obtained before proceeding with the survey activities. Selected individuals were tested for malaria and information collected for any febrile illness in the past 15 days. One team member drew four drops of finger prick blood from each participant. Rapid Diagnostic test (RDT) for malaria was performed on the spot. We also kept one note in our record whether he/she suffered with fever within last 15 days or not. If he/she suffered with fever, we recorded that morbidity. Patients diagnosed as having malaria were provided Coartem for treatment. A socioeconomic survey related to malarial knowledge and relevant health-seeking behavior was also conducted. A semi-structured questionnaire was developed and use for this purpose, the results of which will be presented elsewhere.

### Diagnosis of Malaria

Malaria was diagnosed by Rapid Diagnostic Tests (RDT) that detects both *P. falciparum*-specific antigen and *Plasmodium vivax*-specific antigen. The trade name of this RDT is “FalciVax” and is produced by Zephyr Biomedicals, India (www.tulipgroup.com). Each FalciVax is rapid self-performing, qualitative, two site sandwich immunoassay utilizing whole blood for the detection of *P. falciparum* specific histidine rich protein-2 (Pf, HRP-2) and *P. vivax* specific pLDH. The test can be used for specific detection and differentiation of *P. falciparum* and *P. vivax* malaria. The standardization of this test has already been done by the Zephyr Biomedicals. Sensitivity of the RDT is similar to that commonly achieved by good field microscopy. Sensitivity and specificity of the RDT used for the detection of *P. falciparum* and *P. vivax* is more than 95% and now been recommended for use in the malaria control program by the World Health Organization [12], [13], [14].

### GPS data collections

**Figure 2:**
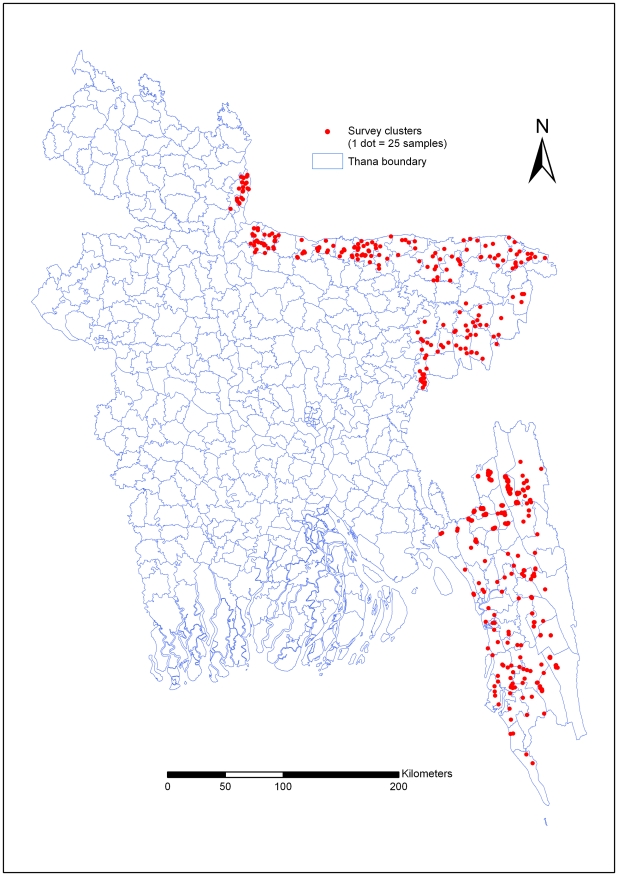
Shows the survey cluster locations.

The coordinates (longitude and latitude) of all selected Mauzas (n = 390) were recorded on-site using eTrex Venture Garmin single handheld GPS receivers. GPS points were uploaded to a Fox Pro database system and checked for accuracy at the field level. GPS points were superimposed on the polygon boundary and verified accuracy. Arc GIS 9.2 software was used for developing map. Figure 2 shows the exact cluster locations from where blood samples were collected.

### Data preparation and statistical analysis

An anonymous database was created and double checked. Fox Pro was used for data entry and storage. Data was cleaned and checked for duplicates. District, Thana, Union, Mauza, household and person identifier was given and later converted into a unique identifier. Of the 9750 questionnaire obtained, one questionnaire about bed nets was lost and 9749 questionnaires were used for analysis. Descriptive summaries of infection prevalence were generated using STATA 10 and MS Excel 2007. To account for the clustered nature of the data, the *svy* command in STATA was used with the cluster as the primary sampling unit (*psu*) stratified by District. All results were weighted (weight = 1/probability of selection) to account for unequal probabilities of selection of clusters across District. To test for differences in proportion of participant livelihood (categorized as south-eastern and north-eastern district), bed-net using status in their house (bed-net related variable is listed as a binary variable with their mean as the cut-off point) between malaria positive and malaria negative household a Pearson chi-square test according to survey design (cluster and stratification) was used and the test statistic converted to an F-statistic using the second-order Rao and Sott correction yielding wider confidence intervals and conservative P-value compared to the uncorrected Chi-square test. To find the independent risk factors for malaria positive households, a logistic regression analysis were performed. Variables with a p value < = 0.05 level in the bivariate analysis (converted F-statistic) were included in a stepwise logistic regression procedure.

## Results

**Figure 3:**
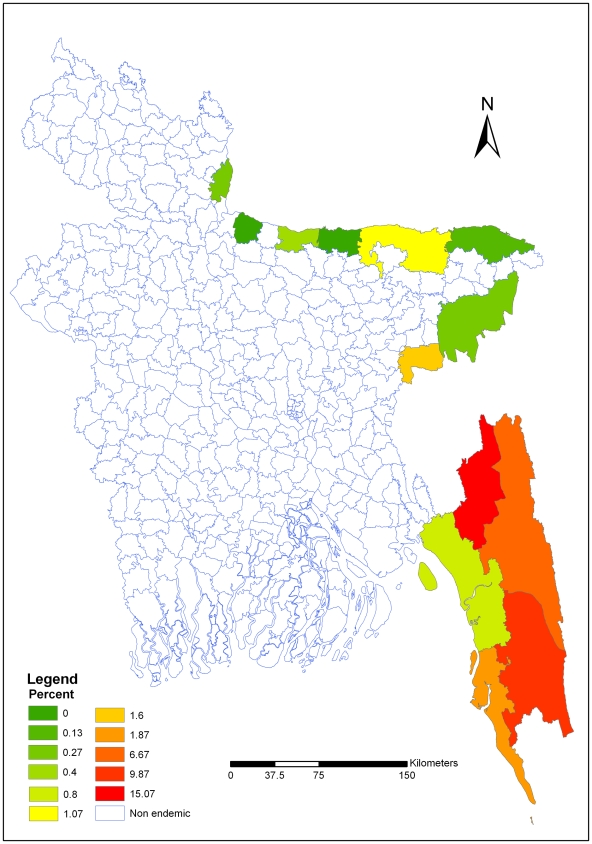
Distribution of *Plasmodium falciparum* in endemic areas of Bangladesh. The distribution of *P. falciparum* is presented in Figure 3. *Plasmodium falciparum* was found in eleven districts of the thirteen surveyed districts. The prevalence rate varied from 0.13% to 15.07%.

**Figure 4:**
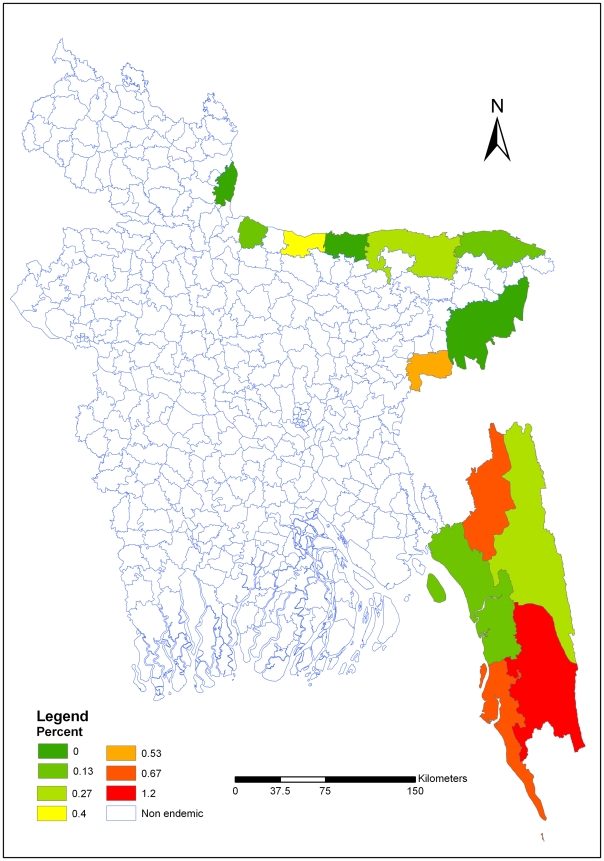
Distribution of *Plasmodium vivax* in endemic areas of Bangladesh. Figure four, shows that *P. vivax* was found in ten districts; in which the prevalence rate varied from 0.13 to 1.2%.

In thirteen malaria endemic districts, the overall weighted malaria prevalence rate was 3.97% (Table 1). The survey clusters (see supplemental information file) and malaria prevalence maps were prepared (figure 2, 3 and 4 respectively). Figure two shows the survey cluster locations. The distribution of *P. falciparum* is presented in figure three. *Plasmodium falciparum* was found in eleven districts of the thirteen surveyed districts. The prevalence rate varied from 0.13% to 15.07%. Figure four, shows that *P. vivax* was found in ten districts; in which the prevalence rate varied from 0.13 to 1.2%. The weighted prevalence of *P. falciparum* was 3.58% and the *P. vivax* 0.21% and mixed infection with *P. falciparum* and *P. vivax* was 0.18%. The proportion of *P. falciparum* was 90.18% while *P. vivax* and mixed infection with these two species were 5.29 and 4.53% respectively in these thirteen districts. Prevalence of *P. falciparum* in males and females was 3.96% and 3.98% respectively. The prevalence of malaria was significantly higher in children. The prevalence of *falciparum* malaria in children 0–4 years of age was 8.5% and 5–14 years of age 6.6%. *P. vivax* prevalence among male are 0.15%, and female are 0.27%. Mixed infection prevalence among male and female were 0.21% and 0.15% respectively. In five south-eastern districts the average weighted prevalence rate was 6.00% while in the eight north -eastern districts average weighted prevalence rate was 0.40%. The overall malaria prevalence in Chittagong Hill Tracts (CHT) districts was 11.7%. The prevalence rate was 15.25%, 10.97% and 7.42% in Khagrachari, Bandarban and Rangamati districts respectively.

**Table 1:** Overall and age, sex and area wise prevalence of different Malaria parasite infection (Weighted).

	Prevalence			
	Any (pf or pv) infection	Pf infection	Pv infection	Mixed (pf and pv) infection
	% [95% CI]	% [95% CI]	% [95% CI]	% [95% CI]
**Over all**	3.97 [3.11–4.83]	3.58 [2.75–4.42]	0.21 [0.08–0.34]	0.18 [0.01–0.35]
**By sex**				
Male	3.96 [2.96–4.97]	3.60 [2.67–4.54]	0.15 [0.01–0.29]	0.21 [−1.1–0.53]
Female	3.98 [2.89–5.08]	3.57 [2.50–4.64]	0.27 [0.06–0.48]	0.15 [0.03–0.27]
**By age group**				
0–4 yrs	11.34 [6.83–15.9]	10.32 [5.98–14.7]	0.63 [−0.03–1.28]	0.40 [−0.26–1.05]
5–14 yrs	8.69 [6.06–11.32]	8.35 [5.75–10.94]	0.21 [−0.04–0.46]	0.13 [−0.02–0.28]
15–49 yrs	2.74 [2.06–3.42]	2.42 [1.77–3.07]	0.19 [0.02–0.37]	0.13 [0.01–0.25]
> = 50 yrs	1.84 [0.89–2.80]	1.30 [0.58–2.03]	0.17 [−0.07–0.43]	0.36 [−0.24–0.96]
**By area/districts**				
South-eastern	6.00 [4.67–7.34]	5.49 [4.18–6.79]	0.31 [0.11–0.49]	0.21 [−0.05–0.50]
North-eastern	0.40 [0.19–0.63]	0.25 [0.01–0.41]	0.04 [−0.04–0.12]	0.11 [0.02–0.21]

When we conducted the survey, we randomly identified one person and performed a RDT. We recorded whether this individual reported fever within last fifteen days. If he/she had suffered with fever within last fifteen days and was RDT positive, we considered that is morbid event. Malaria morbidity was found to be higher in the five south-eastern districts when compared to the eight north-eastern districts.

A total of 9750 participants in the study area, 300 were RDT positive and 9450 were negative. Please see the supplemental information (Table S1). Households of RDT positive participants were considered as malaria infected households and RDT negative participant's households are malaria non-infected households. Bednet use information of 9749 participants was used for statistical analysis and only one participant's information was missing. About 91.1% (n = 267) and 93.7% (8799) participant's households had a bed net among the malaria positive and negative households respectively (p = 0.118, not significant). We have also observed that the bed net use pattern in the infected and non-infected households and found that malaria infected households having significantly less number of bednets (< = 2) compared to malaria non-infected households (Table 2). It was also found that significantly more malaria infected households are in the five south-eastern districts compared malaria non-infected households (Table 2). In the five south-eastern districts the percentage of bed nets treated with medicine within last six months were similar for both types of households. Table 3 shows the adjusted odds ratios (OR) for the variables using the logistic regression model. Independent risk factors for malaria infection found significant in multivariate analysis for the entire surveyed area when less than or equal to two bednets in household (OR = 1.87, 95% CI = 1.30–2.71) and households in south-eastern districts (OR = 17.31, 95% CI = 9.60–31.30).

**Table 2:** Bed net use pattern as well as participants districts by malaria infected and non-infected households (HH).

	Malaria infected HH			Malaria non-infected HH			P-value
	N = 300			N = 9449			
	%	(95% CI)	n	%	(95%CI)	N	
**Bed net status in participant house**							
Having bed net	91.1	(86.6–94.2)	267	93.7	(92.6–94.6)	8799	0.118
Having < = 2 bed nets	57	(49.1–64.6)	198	46.4	(44.4–48.3)	5461	0.006
< = 5 family member use bed net regularly	57.6	(50.0–65.0)	214	55.2	(53.3–57.2)	6487	0.529
Bed-net treated with medicine within last 6 month	01.7	(0.64–5.50)	05	02	(1.3–2.8)	163	0.885
**Last night bed-net use by**							
All family member	80.3	(73.2–86.0)	239	83.8	(82.0–85.4)	7917	0.274
< = 3 adults members	68.1	(61.6–73.9)	232	61.4	(59.2–63.5)	6653	0.044
< = 2 child members	57.4	(48.9–65.5)	206	62.2	(59.7–64.6)	6690	0.255
< = 2 male members	53.3	(46.7–60.0)	191	46.8	(45.3–48.4)	5424	0.054
< = 2 female members	50.5	(43.9–57.2)	189	51.8	(50.1–53.6)	5853	0.885
**Participants districts**							
South-eastern	96.3	(93.5–98.0)	271	62.4	(61.7–63.0)	3480	<0.0001
North-eastern	03.7	(02.1–06.5)	29	37.7	(37.0–38.3)	5969	

**Table 3:** Significant independent risk factors associated with malaria infection: adjusted OR with 95% CI as estimated by logistic regression.

	Malaria infected HH (N = 300)	Malaria non-infected HH (N = 9449)	Adjusted OR	(95% CI)	P- value
**Bed net in house**	**n**	**n**			
< = 2 bed-net	198	5461	1.87	(1.30–2.71)	0.001
>2 bed-net	202	4288	1.00		
**Participants districts**					
South-eastern district	271	3480	17.31	(9.60–31.30)	<0.0001
North-eastern district	29	5969	1.00		
**Bed net in house**	198	5461	1.87	(1.30–2.71)	0.001

## Discussion

In order to implement an effective malaria control program in Bangladesh, accurate information on the incidence and prevalence of malaria is required. In this study, the first malaria prevalence survey was conducted to provide the baseline parasitological information for population living in the malaria endemic districts of Bangladesh. This cross-sectional survey provides point prevalence data on malaria in these thirteen malaria endemic districts. These data will also be a massive help of global initiatives of malaria mapping [15].

We have found a much higher prevalence of malaria than would be expected by investigating the national passive surveillance information [16]. This indicates that by the passive surveillance conducted by the national malaria control program significantly underestimates the burden of malaria in these 13 malaria endemic districts, especially in the five south-eastern districts.

Given that no significant difference was found between male and female malaria prevalence rates, it can be concluded that the gender difference will not be an issue for malaria control. However, there were significantly higher rates of infection among children, thus indentifying a need for targeted interventions for young populations.

Malaria is not equally distributed in all malaria endemic districts of Bangladesh. Prevalence of malaria in five south-eastern districts is significantly higher than the eight north-eastern districts. Chittagong Hill Tracts (CHT) districts have the highest prevalence than the other endemic districts. The reason might be that CHT districts are hilly and covered with forest and lakes, provide an excellent habitat for malaria vectors [17]. Resources should be differentially targeted to this area given its larger malaria burden. The combined use of GIS (geographic information system) and remote sensing provides a significant tool to control malaria [18]. We hope GIS and high resolution satellite images can be used to detect malaria hot spots and vectors habitat sites, particularly in CHT districts, as part of our ongoing work.

From this study it is also clear that *P. falciparum* is the dominant species in the malaria endemic districts of Bangladesh, with the highest prevalence occurring in the CHT districts. This is of high concern since *P. falciparum* is known to be the most deadly and drug resistance for treatment of *P. falciparum* is a worldwide problem [19]. Risk factors regarding the use of bed nets in this study have been found to be consistent with similar studies conducted in Somalia [20]. This study also confirms the results obtained in Vietnam that the households having two or more than two bednets are more protective [21]. The use of two or more than two bednets in a household is an effective malaria control intervention in Bangladesh. Efforts should be focused to increase the supply of at least two bednets in the malaria endemic areas of Bangladesh, especially in the south-eastern districts of Bangladesh.

Total population in endemic areas are 26.9 million, and the risk exposure is as high as in many African countries [22], [23]. Longitudinal studies are needed to assess the variation of asymptomatic parasite carriage over time, and its exact contribution to transmission. Population-based prevalence studies on a regular basis are required to understand the burden of disease. More studies should be conducted in the future to map the changing malaria epidemiology in Bangladesh as control activities are scaled up.

### Limitations

The main limitation of the survey was we failed to start the survey in thirteen districts synchronously. The first the survey started in south eastern part and as soon as we finished, we moved to north eastern part. So, work in the north eastern part was not done during malaria peak season. Any future work mapping the distribution of prevalence in Bangladesh will have to consider this fact in the modelling.

## Supporting Information

Table S1 (0.13 MB XLS)
