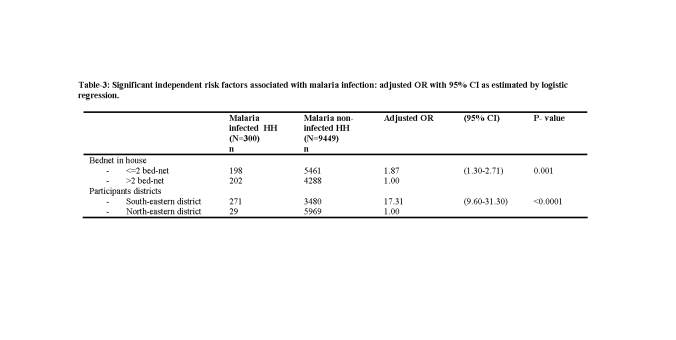# Correction: Malaria Prevalence in Endemic Districts of Bangladesh

**DOI:** 10.1371/annotation/546e7848-4fcb-49d3-8c63-4010445f21da

**Published:** 2009-09-24

**Authors:** Ubydul Haque, Syed Masud Ahmed, Shahed Hossain, Mamun Huda, Awlad Hossain, Mohammad Shafiul Alam, Dinesh Mondal, Wasif Ali Khan, Mohammod Khalequzzaman, Rashidul Haque

The last data row of Table 3 is a repeat of the first. Please view the corrected table here: 

**Figure:**